# High prevalence and low intensity of *Echinophthirius horridus* infection in seals revealed by high effort sampling

**DOI:** 10.1038/s41598-024-64890-z

**Published:** 2024-06-20

**Authors:** Insa Herzog, Ursula Siebert, Kristina Lehnert

**Affiliations:** Institute for Terrestrial and Aquatic Wildlife Research, University of Veterinary Medicine Hannover, Büsum, Germany

**Keywords:** *Phoca vitulina*, *Halichoerus grypus*, Seal louse, Arthropod reproduction, Vector dynamics, Host-parasite interactions, Ecology, Zoology, Diseases

## Abstract

Seal lice (*Echinophthirius horridus*) are bloodsucking ectoparasites of phocid seals and vectors of pathogens like the heartworm, *Acanthocheilonema spirocauda*. Grey and harbour seal populations are recovering in German waters and wildlife health surveillance is crucial for wildlife conservation. A new, high effort sampling protocol for seal lice was applied for grey and harbour seals along the German North- and Baltic Sea coast. Freshly dead seals were systematically sampled within a health monitoring of stranded seals over 12 months. Prevalence, intensity and distribution patterns of seal lice were analysed. 58% of harbour seals (*n* = 71) and 70% of grey seals (*n* = 10) were infected with seal lice. A majority of harbour seals displayed mild levels of infection, while three were moderately and two were severely infected. The head was the preferred predilection site, indicating that *E. horridus* prefers body areas with frequent access to atmospheric oxygen. Nits and different developmental stages were recorded in all age classes in grey and harbour seals in all seasons. For the first time, copulating specimens of *E. horridus* were recorded on a dead harbour seal, highlighting that *E. horridus* reproduces throughout the year on seals of all age classes in German waters.

## Introduction

Harbour seals (*Phoca vitulina*) and grey seals (*Halichoerus grypus*) are top predators in the German North- and Baltic Sea and are considered key species in this environment^1,2^. In the last century both seal species underwent drastic declines due to two Phocine Distemper Virus epidemics, human exploitation and environmental degradation^3–6^. Since the ban of hunting and polychlorinated biphenyls (PCBs) harbour and grey seals are recovering in the North- and Baltic Sea^7,8^. The seal louse (*Echinophthirius horridus*, Echinophthiriidae, Anoplura) is a permanent, hematophagous ectoparasitic insect^9,10^, infecting harbour and grey seals^10–12^. Seal lice are directly transmitted, mainly from adult seals to pups, during haul-outs on land^13,14^. *E. horridus* is assumed to complete its life cycle on seals at shore, with eggs hatching on land^15,16^. The seal louse coevolved with terrestrial ancestors of seals from land into the marine environment millions of years ago^9,17–19^, hence the seal louse plays an important role as potential vector for a variety of infectious pathogens such as viruses and bacteria^20,21^ and heartworms (*Acanthocheilonema spirocauda*) ^11,22–24^. Because prevalence and intensity of ectoparasite infection are important biological indicators for predicting host health and inferring evolution^11,25,26^, precise information on seal louse infection parameters in vulnerable aquatic wildlife are crucial and of parasitological and veterinary relevance^24,27^.The seal louse developed biomechanical and physical adaptations for attachment and respiration in the marine environment^15,28,29^ to sustain high pressure, drag forces, and hypoxemia^30^. The sampling of live lice is relevant for experiments on respiration, to investigate feeding and attachment adaptations^29^ and reproductive processes in vitro.

Apart from the constraints of investigating any protected wildlife species, sampling of marine apex predators includes the challenging environment they inhabit, physiological characteristics of marine mammals and dangers of working with large carnivores^31^. In recent centuries, sampling of seal lice has been based on catching and restraining live animals^13,32,33^ or on immobilisation with sedatives^32–34^. Due to animal welfare considerations, less invasive methods with a “telescopic lice comb apparatus” enabled sampling from a distance without restraining the animal, but were limited by the reliance on small sample sizes of synanthropic South American sea lions (*Otaria flavescens*), which are habituated to the presence of humans^35^. Other methods involved restraining juvenile South American sea lions while combing the animal and collecting lice but without sampling the head area^36^. Quantitative lice collection on live pinnipeds remains logistically challenging and incomplete. *Post-mortem* investigations however, are useful for recording a variety of health parameters, providing information about the cause of death^37–40^, including ectoparasites and associated lesions^24^. Patterns of louse distribution on their pinniped host contribute to understanding the adaptations of a long co-evolutionary relationship between parasite and host, allowing both parties to survive^41^. The detection of nits and developmental stages is crucial for decoding the reproductive strategies of this marine insect, adapted to reproduce on diving hosts, which can spend weeks or months at sea^42^.

*E. horridus* prevalence was previously investigated in the frame of *post-mortem* sampling in the North- and Baltic Sea^11,24,43^ and in North American waters^44^. Although the seal louse prevalence in the Wadden Sea, Northern Europe was low after the Phocine Distemper Virus epidemics^11,24,43^, the prevalence at the Washington Coast, USA was 45%^44^. Ectoparasite records are often biased, as parasites typically leave their host actively after the death of their host^25,33^ or are lost after death during drifting at sea, the stranding event, scavenging, or during transport and storage until the necropsy^11,24^. Intensity of *E. horridus* infections was recorded semi-quantitatively during *post-mortem* investigations^11,24^ and distribution patterns of seal lice have not been systematically reported. Therefore quantitative data on seal lice infections are scarce, and little knowledge exists on their predilection sites on grey and harbour seals. The aim of this study was to systematically record prevalence and intensity of seal lice by applying a personnel-intensive strategy to reduce sampling bias in ectoparasite records and to obtain infection parameters as close to *in vivo* ectoparasite infection as possible. In this study, a novel *post-mortem* sampling strategy for *E. horridus* revealed new insights into seal louse prevalence, intensity and distribution patterns.

## Materials and methods

### Study area and sampling

**Figure 1:**
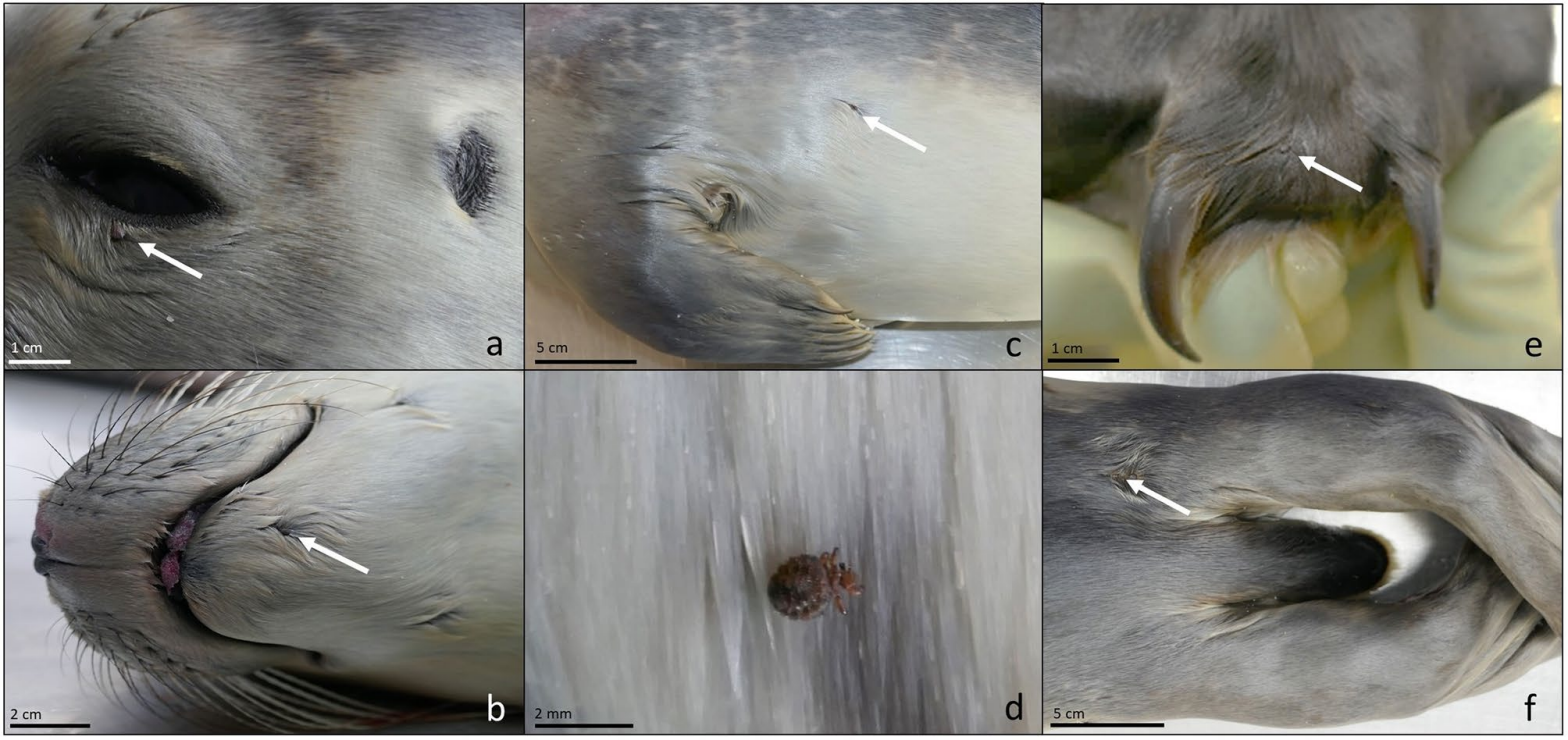
Different infected body parts in juvenile harbour seals. (**a**) *E. horridus* located close to the eyelid. (**b**) *E. horridus* infection in the muzzle area. (**c**) *E. horridus* infection in abdominal region. (**d**) Close- up image of live *E. horridus* moving on harbour seal fur. (**e**) *E. horridus* located on fore flipper, between harbour seal claws. (**f**) *E. horridus* infection of hind flipper. a-f, White arrows pointing at *E. horridus*.

Seventy-one harbour seals and ten grey seals, collected within the stranding network of the federal state of Schleswig–Holstein (SH) along the North- and Baltic Sea coast from April 2022 until April 2023, underwent a newly developed, personnel-intensive examination. Seals included in this study were either found freshly dead or mercy killed by a licensed seal ranger. Carcasses were transported in a sealed plastic bag to the Institute for Terrestrial and Aquatic Wildlife Research (ITAW) in Büsum. One veterinarian systematically screened all animals for ectoparasites. Before examination, blood and organic contaminants were removed by washing with water to gain clear view of the fur. Body areas were categorised in body area 1: head, body area 2: fore or/and the hind flippers, tail, body area 3: head and fore and/or hind flippers, tail, and body area 4: complete body of the animal/ no clear distinction of infection possible. The seals were combed with a commercially available louse comb for human head lice (Dirk Rossmann GmbH, 2022). The direction of combing was parallel to the direction of the fur, which provided a smooth surface. The sleek fur coat of the carcass was then examined for small knoblike irregularities displaying the presence of seal lice (Fig. 1). All lice specimens, nits and developmental stages were removed with a louse comb or forceps and preserved either in ethanol (70%), formalin (10%) or were frozen. Nymph stages 1–3 were morphologically identified according to Leidenberger et al.^10^. Lice specimens were counted and the infected body area was recorded. Lice counts were additionally grouped into levels of infection classified as mild infection, < 20 lice; moderate infection, 20–39 lice; and severe infection, ≥ 40 lice. Seals were allocated to different age groups, young-of-the-year seals, AG 1 (up to 6 months), yearlings, AG 2 (7–18 months) and adults, AG 3 (> 18 months). After the examination for seal lice, a complete *post-mortem* investigation after a standardized protocol^37–39^ was performed. All host animals in this study were found dead, died naturally or were euthanized based on welfare grounds and in accordance with the state hunting law of Schleswig–Holstein, Germany. None of the host animals were euthanized or killed for the purpose of this study. No consent from an Animal Use Committee is required when dealing with dead animals, as was the case here. Consequently, animal ethics committee approval was not applicable to this work. All methods were carried out in accordance with relevant guidelines (Ministry for Energy Transition, Climate Protection, Environment and Nature, Schleswig–Holstein, Germany) and in accordance with ARRIVE guidelines.

### Statistical analysis

Prevalence and intensity of infection were recorded according to Bush et al.^45^. Prevalence and statistical significance were calculated using the software Quantitative Parasitology (QPWeb, V 1.0.15)^46^. To test the independent variables “sex” (male, female), “infected body area” (1–4) and “age groups” (AG1, AG2, AG3) in terms of significant differences in intensity (dependent variable) a Kruskal–Wallis test was performed. To determine in which groups differences in regard to the dependent variable can be observed, Dunn´s test with Bonferroni correction was performed, and the effect size was calculated by r = $\frac{z}{sqrt(N)}$^47^. Significance level was set at 0.05. Statistical analysis regarding intensity was carried out using R 4.2.1(R Core Team, 2021) using the *dplyr*^48^, *rstatix* (*v0.7.0*;^49^ and *ggplot 2* (*v 3.4.1*;^50^) packages.

## Results

### Prevalence, intensity and distribution patterns

58% (n = 41) of the investigated harbour seals (n = 71) were infected with seal lice. 68% (n = 25) of male harbour seals (n = 37) and 47% (n = 16) of female harbour seals (n = 34) displayed a seal louse infection. 57% (n = 31) of the young-of-the-year seals (n = 54), 69% (n = 9) of the yearlings (n = 13) and one adult harbour seal (n = 4) were infected. No significance in prevalence between sexes (Fisher´s exact test, *P* = 0.09) or age groups (Fisher´s exact test, *P* = 0.37) was detected.

**Figure 2:**
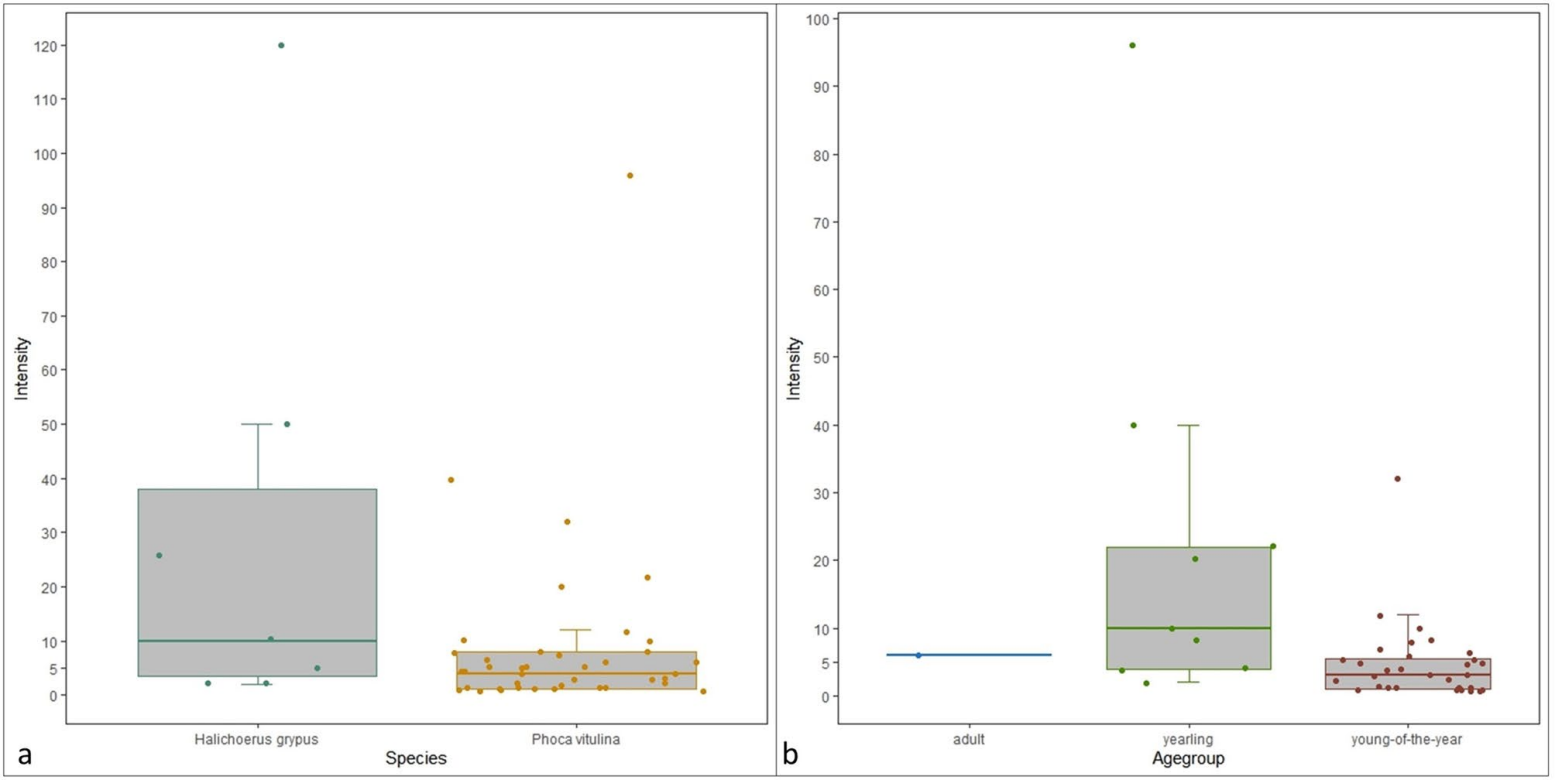
Intensity of *E. horridus* infection in different groups*.* (**a**) Intensity of infection with *E. horridus* in harbour and grey seals from the North- and Baltic Sea (from April 2022 until April 2023). (**b**) Intensity of infection in different age groups of harbour seals.

**Figure 3:**
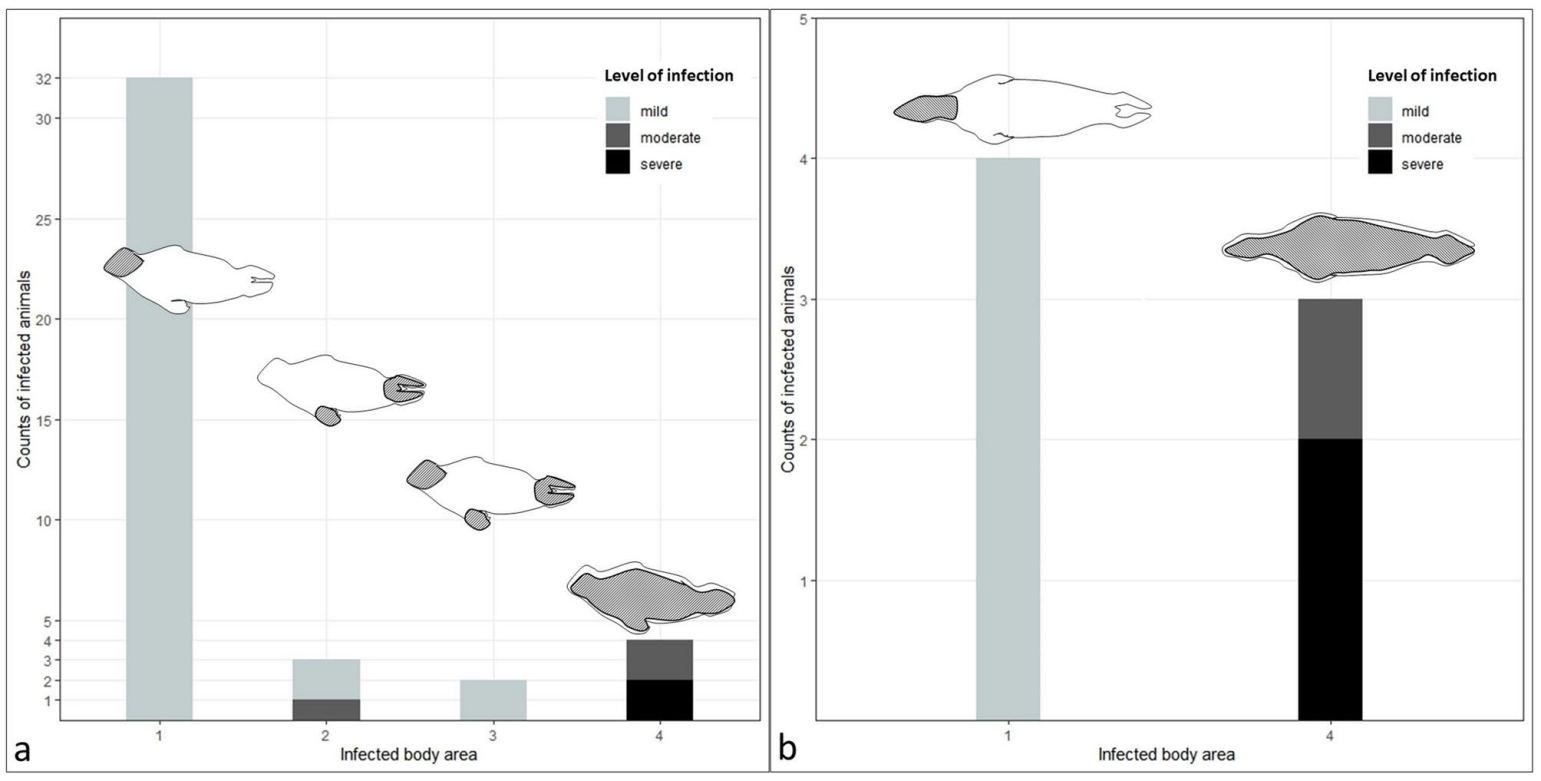
Levels of *E. horridus* infection and distribution patterns in grey and harbour seals. (**a**) Infected body areas 1–4 and levels of infection in harbour seals from the North- and Baltic Sea (from April 2022 until April 2023). (**b**) Infected body areas 1 and 4 and levels of infection in grey seals from the North- and Baltic Sea (from April 2022–April 2023). Pictograms represent the species and dark-grey shaded areas symbolize infected body areas.

Intensity of lice infection in harbour seals ranged from 1 to 96 lice (Fig. 2). Thirty-six harbour seals were mildly infected, three were moderately infected, and two were severely infected. Significant differences in intensity were observed between body areas (Kruskal-Wallis test, *X*^2^ = 10.87, *df* = 3, *P* = 0.01). Body area 1 (head) differed significantly in intensity from body area 4 (complete body) (Dunn's test with Bonferroni correction, *Z* = 3.27, *P* = 0.001, *P.adj* = 0.006, *r* = 0.54). In this study, body area 1 was most often infected with seal lice (78%). All harbour seals that were only infected within body area 1 were mildly infected (Fig. 3). Harbour seals that showed an infection in body area 4 were severely (n = 2) or moderately (n = 2) infected. No significant difference in intensity was observed between other body areas. Intensity differed significantly between age groups (Kruskal-Wallis test, *X*^2^ = 8.1139, *df* = 2, *P* = 0.02). Significantly higher intensity of seal louse infection was found in yearlings compared to young-of-the-year-seals (Dunn's test with Bonferroni correction, *Z* = 2.78, *P* = 0.005, *P.adj* = 0.02,* r* = 0.44) (Fig. 2). No significant difference in seal louse infections was detected between male and female harbour seals (Kruskal-Wallis test, *X*^2^ = 0.42, *df* = 1, *P* = 0.52).

70% (n = 7) of grey seals (n = 10) were infected with seal lice. Five out of six male grey seals were infected, while two out of four females were infected.

Two out of two young-of-the-year grey seals were infected with seal lice, one yearling (n = 3) and four of the adult grey seals (n = 5) were infected. No significant differences were observed in prevalence between different sexes (Fisher´s exact test, *P* = 0.5) or age groups (Fisher´s exact test,* P* = 0.33).

In grey seals, seal lice intensity ranged from 1 to 120 lice (Fig. 2). Four grey seals displayed a mild infection, one animal was infected moderately and two were infected severely. Infections were only observed in body area 1 (head) and body area 4 (entire body) (Fig. 3). A significant difference in intensity between those body areas was observed (Kruskal-Wallis test, *X*^2^ = 4.58, *df* = 1, *P* = 0.03). Intensity did not differ significantly between male and female grey seals (Kruskal-Wallis test, *X*^2^ = 0, *df* = 1, *P* = 1) or between different age groups (Kruskal-Wallis test, *X*^2^ = 3.8182, *df* = 2, *P* = 0.15).

No significant difference in prevalence (Fisher´s exact test, *P* = 0.52) or intensity (Kruskal-Wallis test, *X*^2^ = 2.92, *df* = 1, *P* = 0.09) between harbour seals and grey seals was observed.

### Reproduction of *E. horridus*

Nits and nymph stages 1–3 of *E. horridus* were found on harbour and grey seals and on males and female seals of all age classes. Nits and developmental stages occurred throughout the sampling year in all seasons (see Table 1, Supplementary Data 1).

**Table 1:** Nits and developmental stages of *E. horridus* on grey and harbour seals recorded from April 2022 until April 2023.

ID	Marine mammal species	Age group	Sex	Sampling month	Level of infection	Nit	Nymph 1	Nymph 2	Nymph 3
P.v. 1	*Phoca vitulina*	2	Male	4	Severe	+	+	+	+
P.v. 2	*Phoca vitulina*	3	Male	4	Mild	−	−	+	−
P.v. 3	*Phoca vitulina*	1	Female	8	Moderate	−	−	+	+
P.v. 4	*Phoca vitulina*	1	Male	9	Mild	+	−	−	−
P.v. 5	*Phoca vitulina*	2	Female	4	Severe	+	+	+	+
P.v. 6	*Phoca vitulina*	1	Female	2	Mild	+	−	−	−
H.g. 1	*Halichoerus grypus*	3	Male	11	Severe	+	+	+	+
H.g. 2	*Halichoerus grypus*	2	Female	2	Moderate	+	−	+	+
H.g. 3	*Halichoerus grypus*	3	Male	10	Severe	+	+	+	+

Symbols explained: +  = present;  − = absent.

**Figure 4:**
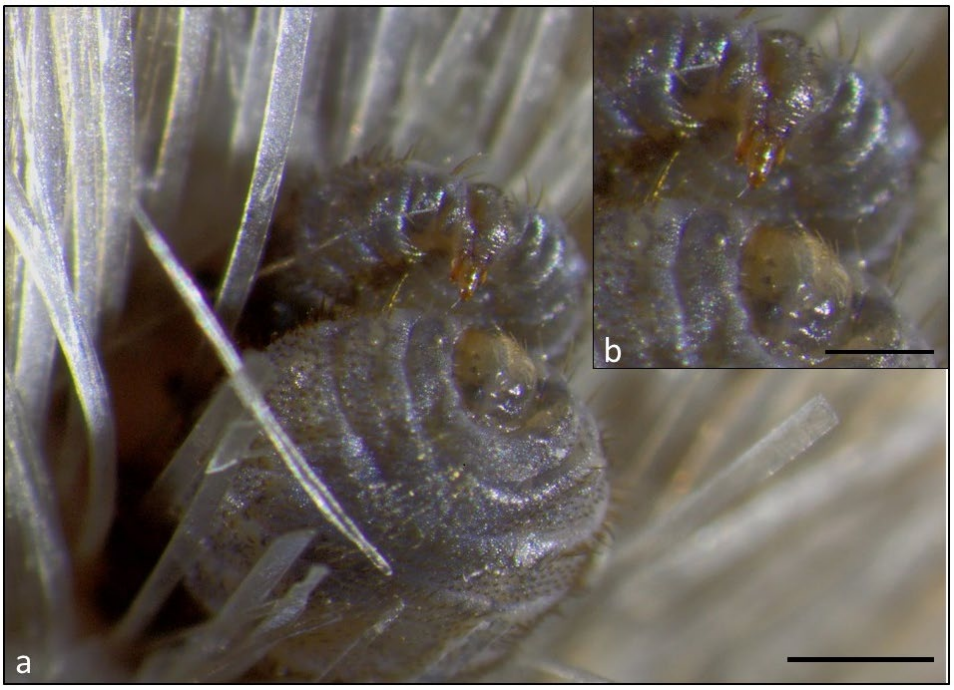
Reproduction of *E. horridus*. (**a**) Female and male *E. horridus* during copulation on a yearling harbour seal. (**b**) Close-up of male genitalia. a, b: bars: 1 mm.

In April 2023, two adult live lice were observed during copulation (Fig. 4) on a female yearling harbour seal from the North Sea, approximately 12 hours after the animal was mercy killed. The two specimens were subsequently preserved in ethanol (70%). The male louse was attached to the hair and skin of the seal´s head. The dorsal site of the abdomen of the male louse was placed underneath the ventral abdomen of the female louse. The abdomen of the male was dorsally bent with the male genitalia pointing towards the genital region of the female. The female louse was attached to the hair shafts of the seal.

## Discussion

In this study, the prevalence of seal lice in harbour and grey seals in the North- and Baltic Sea are considerably higher than that reported in previous studies. In the same geographical study area, a notably lower prevalence of 3.4% in harbour seals was recorded between 1996 and 2013^11^, and 4% in harbour and 10% in grey seals were recorded between 2014 and 2021^24^ based on *post-mortem* investigations. In 1971/72 a prevalence of 41% was recorded for mainly hunted harbour seals in the Wadden Sea in Lower Saxony, Germany^51^. No seal louse infection was observed in dead harbour seals from the Kattegat-Skagerrak and the Baltic region after the first Phocine Distemper Virus epidemic^43^. Studies based on sampling of live animals in Scottish waters found a prevalence of 39% in the local harbour seal population^33^.

Results highlight standardized, detailed and comprehensive visual examination of freshly dead aquatic mammals to be an effective method to determine prevalence of permanent ectoparasites^25,26^, including the marine insect *E. horridus.* The striking difference with regard to previous *post-mortem* studies of the North- and Baltic Sea presumably depends on the high effort sampling protocol. In the present study, only freshly dead animals were examined, and loss of ectoparasites due to scavengers, drifting at sea, storage and freezing of the carcass was limited. Systematic and detailed screening carried out by trained personnel is crucial to detect seal lice, which are well camouflaged and remain strongly attached to their host. Nevertheless, possible *post-mortem* migration of parasites could influence the observed distribution patterns and intensity. While ectoparasites are known to quickly leave their dying host^25,52^, in small terrestrial mammals, lice were observed to leave their host later than ticks and fleas^53^. The results of this study contradict previous assumptions that seal lice might leave their host immediately after its death and remain in the haul-out substrate^33^. Rather, this indicates that seal lice stay on their host, unless another suitable host is in accessible proximity. Sick or weak animals are often unable to return to the water after haul-out sessions^54^, hence remain isolated on land^55^; consequently, lice are forced to remain on their host. Additionally, in weakened host individuals self-grooming might be limited and thus the ectoparasite load increases^56,57^. However, recovering harbour and grey seal populations and higher densities of seals on shared haul-out sites may also facilitate interspecies and intraspecies transmission and higher prevalence^24^.

This study highlights that seal louse prevalence in harbour and grey seal populations in the North- and Baltic Sea is much higher than previously assumed. Seal louse infections are widespread, but levels of infection are predominantly mild and limited to few parasite individuals per host. This aggregated distribution of parasites in natural host populations has been observed for many parasites^58,59^, most host individuals display a mild or no parasitic infection, while only a few host individuals are severely parasitized.

The possible role of *E. horrdius* as vector of *A. spirocauda* remains a central object regarding the importance of seal louse prevalence. Microfilaria were found in blood smears of 41% of wild-caught seals sampled from 2008 to 2014^11^, and the present study reportes similarly high prevalence of *E. horridus* in the same geographical area*.* This finding supports *E. horridus* as possible vector for the filarial heartworm, *A. spirocauda*^24^. Contrary, prevalence of adult heartworms located in the heart has been considerably lower^11,24^. The death of adult female nematodes after the release of microfilarial stages into the blood, called ephemerality, is common in other filarioid nematodes, in which production of microfilaria is minimized to a certain amount of time, preventing vectors from continuously ingesting microfilaria^60^.

In this study, the head was the body area most often infected with seal lice. In all observed cases, in which only the head area was infected, the level of infection was mild. If animals were severely infected, the entire body was infected. Results indicate the head as preferred body area, while the rest of the body is parasitized when the space around the head becomes limited due to increasing population size of the parasite on its host. Distribution patterns of ectoparasites on hosts are mainly determined by the microhabitat most suitable for the parasite, e.g. in terms of grooming and differing skin thickness^61^. In birds, lice parasitize areas of the body that are difficult for the animal to reach while preening^25,62^. *E. horridus* has also been suggested to infect body parts difficult to access for their host^63^. Due to the anatomical characteristics like highly specialized and motile fore flippers^64,65^, seals are able to groom all parts of their head. During movement on land, the ventral abdomen and flippers are mechanically stressed, which could prevent ectoparasites from infecting those areas^16^. During periods at sea, the seal´s head is most frequently above the water surface and in contact with atmospheric oxygen, crucial for seal lice respiration. For *Antarctophthirus callorhini*, and *Proechinophthirus fluctus,* seal lice found on Northern Fur Seals (*Callorhinus ursinus*), surface temperatures of 25–35 °C were considered optimal for development of the larval stages^17^. In harbour seals, the surface temperature of the head ranges around 15 °C, while the snout in particular accomplishes surface temperatures up to 20 °C^66^. The high vascularization of the sensory tactile system^67^ and the reduced blubber thickness in the head area^68^ could additionally benefit the feeding process of the hematophagous insect, providing a small distance between body surface and vessels, allowing seal lice to feed and to complete their lifecycle.

In particular in juveniles, infection of the head area could be an indication of the transmission pathway of *E. horridus*. The harbour seal mother keeps nose-to-nose contact continuously within the first minutes *post-partum*^69^. Nose-to-nose, nose-to-body and contact during suckling are maintained in the following weeks during nursing^70^, thus the head could be the area most likely to be infected first. For other seal lice species, infections of pups are observed hours after birth^14,17^. It should be noted that *E. horridus* was sampled from adult harbour seals but the sample size remains low compared to other age classes.

In single cases, infections of the head area with *E. horridus*^71,72^ were described, while severe levels of infection were present on the ventral surface of neck, sacral and genital regions in harbour seals^73^. A systematic study based on *E. horridus* infected live harbour seals on Scottish shores suggested that the primary infection pattern was the hind flippers^33^, similar to infection patterns found in otariid seals^32,74^, although the entire body was not sampled. Higher prevalence of eggs and ovigerous female lice (*Antarctophthirus microchir*) on the dorsal back compared to the ventral belly was reported for South American Sea lion pups (*O. flavescens*), while for nymphs and male *A. microchir*, the opposite pattern was recorded. However, only limited conclusions are possible as the head, neck and fore flippers were not examined^16^.

Data on the reproductive cycle of *E*. *horridus* is scarce. In this study, nits were found on young-of-the-year harbour seals as well as on yearling seals in all seasons. In grey seals, nits and nymphal stages were sampled from a yearling grey seal and two adult grey seals. Seal lice reproduced on a yearling harbour seal in April. Findings of the current study indicate that *E. horridus* does reproduce independently of grey and harbour seal reproductive behaviour. Contrary to this finding, it was assumed that seal lice species *Antarctophthirus microchir* on South American sea lions (*O. flavescens*) synchronize their reproduction with that of their host^14,75,76^. On pups, which spend sufficient time on land, seal lice are able to reproduce and complete their nymphal development^16,77,78^. Findings from this study contrast with reports of seal lice infecting pinnipeds in the Southern hemisphere (e.g. *A. microchir*) which are suggested to produce only one to two lice generations per year during the pupping season of their host^14,77^. In the Northern hemisphere, *E*. *horridus* infects two phocid seal species with different parturition characteristics and lactation times. While harbour seals give birth from May to August, grey seals are born during winter (November–February)^79^. While harbour seals shed their fetal pelage prenatally, grey seal pups are born with lanugo and undergo their first moult approximately 3–6 weeks *post-partum*^79^. Harbour seal pups are able to swim with their mothers within hours after birth^80^, while grey seal pups remain on land for their lactation period of approximately 19 days and in some populations even during their fasting period after weaning^81^. When *E. horridus* is transmitted to a grey seal pup, it needs to survive the impediment of the lanugo fur moult, but the time on land is extended compared to that of harbour seal pups. Our results highlight that the ectoparasitic insect *E. horridus* has adapted to different and challenging reproductive behaviours of two seal species and has evolved to complete its life cycle successfully on a marine host.

## Conclusion

Novel quantitative data about seal lice prevalence, intensity, and infection patterns are reported for two phocid hosts. New information about nits and developmental stages of seal lice on grey and harbour seals was collected from all age classes and the entire seal body for the first time. The findings underline the importance of performing profound and systematic *post-mortem* investigations within a fast and efficient marine mammal stranding network, essentially relying on quick communication between wildlife researchers and seal rangers and other non-scientific helpers. Ectoparasites represent an important indication of ecological relationships, highlighted by increasing prevalence of *E. horridus* corresponding with recovering population sizes of harbour and grey seals. Higher seal numbers on haul-out sites enable inter- and intraspecific ectoparasite transmissions. Seal lice as potential vectors for multiple pathogens need to be monitored with regard to environmental changes, which can accelerate host-parasite interactions and facilitate ectoparasite transmission.

### Supplementary Information

Supplementary Information.

## Data Availability

All relevant data collected during this study are included in the article and provided as supplementary data.
